# Attention Bias Test Differentiates Anxiety and Depression in Sheep

**DOI:** 10.3389/fnbeh.2018.00246

**Published:** 2018-10-23

**Authors:** Jessica E. Monk, Sue Belson, Ian G. Colditz, Caroline Lee

**Affiliations:** ^1^Agriculture and Food, Commonwealth Scientific and Industrial Research Organisation, Armidale, NSW, Australia; ^2^School of Environmental and Rural Science, University of New England, Armidale, NSW, Australia; ^3^Sheep CRC, University of New England, Armidale, NSW, Australia

**Keywords:** affective state, animal welfare, behavior, cognitive bias, emotion, stress-induced hyperthermia, threat perception, vigilance

## Abstract

Negative affective states such as anxiety and depression pose a risk to animal welfare, however, practical tests for assessing these states in animals are limited. In humans, anxious individuals are shown to pay more attention toward threatening information than non-anxious individuals, known as an attention bias. Previously, an attention bias test was developed and validated as a measure of anxious states in sheep, where more anxious sheep showed increased attention toward a threat (dog) and were more vigilant than Control animals. Studies in humans suggest that attention biases also occur in depressed individuals, with observations of attention biases toward threats, as well as biases away from positive stimuli. Given these findings, we hypothesized that an attention bias test for sheep could also be used to assess states of depression. We predicted that Merino ewes in pharmacologically induced Depressed (para-chlorophenylalanine) and Anxious (m-chlorophenylpiperazine) states would show greater attention toward a threat than Control animals (saline), but that the Depressed sheep would show relatively less interest in a positive stimulus (photograph of a conspecific). During testing, Depressed sheep paid more attention toward the threat and less toward the photograph than Control animals as predicted (Analyses of Variance, *P* < 0.05, *n* = 16 per treatment). Interestingly, Anxious sheep showed an attention bias in the opposite direction, paying more attention toward the photograph and less toward the threat than Control animals (*P* < 0.05). Both Anxious and Depressed sheep were more vigilant than Control animals (*P* = 0.002). These results suggest the attention bias test can be used to measure and differentiate states of depression and anxiety in livestock. The bidirectional nature of the attention bias identified between treatments highlights the importance of measuring multiple behaviors in the test and considering the context in which the test is applied. This will enable a clearer characterization of the affective state of an animal, as an aspect of its welfare.

## Introduction

The affective states of animals comprise an important component of animal welfare, with negative depression-like states (hereafter depression) and anxiety-like states (hereafter anxiety) potentially posing a risk to the well-being of livestock in production systems. Practical methods which can assess these negative affective states would therefore be useful for the study, assessment and improvement of animal welfare. Cognitive methods from the human literature offer the potential to assess a range of different affective states in animals (Paul et al., 2005). For example, it has been well established that humans in anxious states pay more attention toward threatening stimuli than non-anxious individuals, known as an attention bias (Bradley et al., 1995, 1997; Bar-Haim et al., 2007). Based on this concept, a test has been developed for assessing attention biases in sheep, measuring the degree to which an animal’s attention is directed toward a potential threat (location recently occupied by a dog) and away from a positive stimulus (food). The test has been validated as a measure of anxious states, where animals in a pharmacologically induced anxious state spent more time looking toward the previous location of a dog, were more vigilant and were less likely to feed than calm (saline control) animals during a 3 min test (Lee et al., 2016; Monk et al., 2018). While attention biases toward threats have predominantly been associated with anxious states in the human literature, there is some evidence that attention bias toward threats also occurs in depressed individuals (Mogg et al., 1995; Mathews et al., 1996), although this has not been consistent in all studies (see reviews Peckham et al., 2010; Armstrong and Olatunji, 2012). However, evidence of attention biases away from positive stimuli occurring in depressed individuals is more consistent (Armstrong and Olatunji, 2012). Given these findings, we might then expect that states of anxiety and depression in animals can both result in attention biases toward threats, while depression can result in a bias away from positive stimuli, when compared to normal individuals. The potential for an attention bias test to assess not only anxious states, but also other negative affective states such as depression in livestock deserves further investigation.

Anxiety and depression in human and animal models are also associated with different sets of behavioral and physiological responses, which may help to differentiate these states during an attention bias test. For instance, key features of anxiety in humans include increased fearfulness of novel experiences or environments and exaggerated fear responses to potential threats (Guze, 1995). These features can be measured in animals through behaviors such as reduced interactions with the novel environments and changes in locomotion (see reviews Boissy, 1995; Forkman et al., 2007; Dodd et al., 2012). During the attention bias test for sheep, this was also reflected by increased vigilance in anxious animals (Lee et al., 2016, 2017; Monk et al., 2018). A key feature of depressive states in humans is a loss of interest or pleasure in daily activities (anhedonia) (Guze, 1995), which has also been reproduced in rodent models of depression (e.g., Moreau et al., 1992; Barr and Phillips, 1999; Harrison et al., 2001). During an attention bias test, this may be reflected by reduced interactions with the positive stimulus. Physiological responses during testing may also help to differentiate anxious and depressed states. For example, stress-induced hyperthermia (SIH) is shown to be reduced by anxiolytic but not anti-depressant drugs (Bouwknecht et al., 2007) and has been previously observed in anxious cattle during an attention bias test (Lee et al., 2017). By considering these key differences in behavioral and physiological responses along with the presence or absence of an attention bias toward the threat, it may be possible to differentiate states of anxiety and depression in sheep during an attention bias test.

Controlling external factors which can influence behavior in the attention bias test is essential for the standardized application of the test and effective interpretation of behavioral responses. The previously established method relies on a feed reward as a positive stimulus, however, motivation to feed can vary considerably between animals and within animals over the span of a single day. Further, while pharmacological models of affective state provide a useful tool for validation of test methodology, these agents have known impacts on appetite, making interpretation of results difficult (Doyle et al., 2015). As such, it would be desirable to replace the feed reward used in the established test method with an alternative positive stimulus that can be more easily standardized and allow for clearer interpretation of behavioral responses. As sheep are social animals, a conspecific could work as an alternative positive stimulus, however, addition of another live animal could again introduce unwanted variation into the test. Photographs of conspecifics have been shown to reduce fear-related behaviors of isolated sheep in previous studies, indicating they were perceived as positive by the test animals (Vandenheede and Bouissou, 1994; Bouissou et al., 1996). As such, a photograph or model of a conspecific may be a more suitable alternative positive stimulus than food for the attention bias test which can be presented in a more standardized manner during testing.

The aim of the current study was to determine whether responses in the attention bias test for sheep may indicate affective states of depression as well as anxiety. We hypothesized that sheep in pharmacologically induced states of depression and anxiety would both show an attention bias toward the threat relative to the control animals. Further, we hypothesized that it would be possible to differentiate between states of depression and anxiety by comparing their relative attention toward the positive stimulus and by considering other behavioral and physiological responses in the test. Specifically, we expected the two treatment groups would differ in temperature response and activity during the test as well as exploration of the positive stimulus and environment. Finally, we hypothesized these attention biases could be assessed using a photograph or model of a conspecific as a positive stimulus instead of the feed used in previous studies. To test these hypotheses, states of depression and anxiety were induced through pharmacological manipulation of serotonergic pathways prior to assessment in a modified attention bias test.

## Materials and Methods

### Animal Ethics

The protocol and conduct of the experiments were approved by the CSIRO F.D. McMaster Laboratory Animal Ethics Committee (AEC17-25) and the University of New England Animal Ethics Committee (AEC17-126), under the New South Wales Animal Research Act 1985.

### Pilot Study

A pilot study was conducted to determine an appropriate positive stimulus to replace food in the attention bias test. A fiberglass model of a sheep and a range of photographs were trialed (see below for details).

#### Animal Details

Fifteen, non-pregnant, non-lactating Merino ewes (2.5 years old) from the CSIRO farm flock in Armidale, NSW were used in the pilot study; five sheep during Phase 1 of the experiment and ten sheep during Phase 2 of the experiment. Sheep had no prior experience with the attention bias test or the positive stimuli used in this trial. Sheep had prior experience with dogs during routine farm management.

#### Attention Bias Test Arena

**FIGURE 1:**
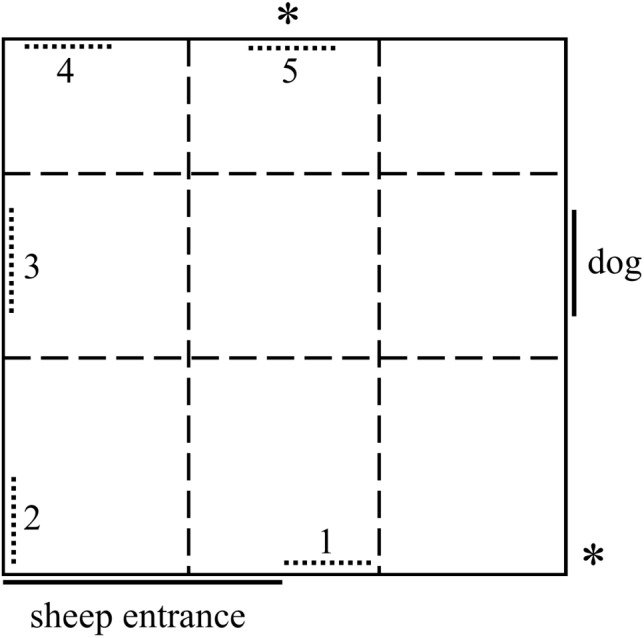
Diagram of the attention bias test arena comprising a 4 m × 4.2 m yard with opaque 1.8 m high walls. A dog was visible for the first 3 s of the test, then the window was covered. The numbered, dotted lines represent potential locations of positive stimuli during the pilot study. Only position 3 was used during the main trial. “^∗^” Denotes the positions of two cameras. Dashed lines represent a grid overlaid on video footage post-testing.

The current study used the same testing arena (Figure 1) as Monk et al. (2018), which was adapted from Lee et al. (2016). The test arena comprised a 4 m × 4.2 m concrete yard with 1.8 m high opaque walls. A small window was positioned on the side of the arena behind which an unfamiliar dog (Border Collie) was standing quietly. Alternative positive stimuli could potentially be placed along the walls opposite and adjacent to the dog window (Figure 1, see section “Alternative stimuli” for details). The test arena was divided into a grid with nine sections, which were overlayed on the video footage during behavioral analysis (Figure 1). Due to constraints of the arena construction material, the dog window was not centered along the side wall. As such, the grid was skewed so that the middle zones remained centered around the dog window (Figure 1). When a sheep was introduced to the arena, the dog was visible through the window for 3 s, after which the window was covered by a retractable opaque cover and the dog was removed. Exposure for 3 s was previously shown to be sufficient to affect attention behaviors in sheep (Monk et al., 2018). The 3 s interval began once the door into the arena was closed behind the sheep and a hidden observer was confident that the sheep had made visual contact with the dog. The hidden observer was positioned above the arena in a building next to the test arena, out of view of the sheep. The 3 min test interval commenced once the window was fully closed to obscure the dog. Behaviors during the test were recorded by a Sony Handycam handheld video camera (Sony, Australia, model number HDR-XR550) (Figure 1).

#### Alternative Stimuli

**FIGURE 2:**
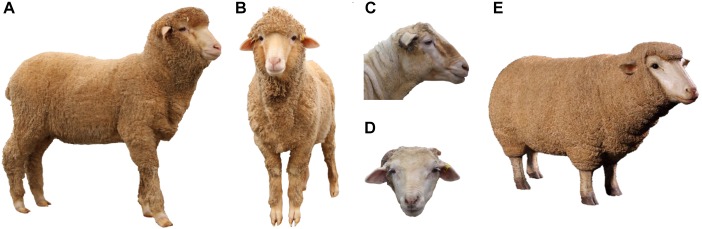
Alternative stimuli trialed during the pilot study. Stimuli included photographs of entire sheep from the side **(A)** and front **(B)**, photographs of faces only from the side **(C)** and front **(D)** and a three-dimensional fiberglass model of a sheep **(E)**. All stimuli were approximately life-size.

To replace the feed reward used in the original attention bias test, five alternative stimuli were trialed during the pilot study: a fiberglass model of a sheep, two photographs (hereafter photo) of an unfamiliar Merino sheep (one side profile, one front-on), and two photos of the faces of an unfamiliar Merino sheep (one side profile, one front-on) (Figure 2). The model and photos were approximately life-size. All photos were printed on 200 gsm matte cardstock, then were cut out and mounted to 5 mm thick black corflute board using spray adhesive. The front-on head only photo had a small amount of wool glued to the top of the head as an additional sensory cue. The wool came from an unfamiliar conspecific, collected from a shearing shed.

#### Phase 1: Identification of an Alternative Positive Stimulus

Phase 1 of the pilot study aimed to identify which of the alternative stimuli was preferred by the sheep. Animals were first tested as a group, then individually during an attention bias test.

A group of five sheep had numbers painted on their rumps for individual identification, then were moved into a small yard (5 m × 4 m) containing all five stimuli for 30 min. The stimuli were located along the edges of the yard, spaced approximately 2 m apart from one another. The number of interactions each sheep had with each of the stimuli was recorded. Interactions included sniffing or attempting to chew the stimuli. Sheep were not exposed to a dog during this time.

All five stimuli were then moved into the attention bias test arena together. The locations of the stimuli in the arena are shown in Figure 1; the stimuli were spaced approximately 1–2 m apart. Each of the five sheep individually underwent the attention bias test as described above, including exposure to a dog for 3 s. During the 3 min test, the number of interactions sheep had with each of the alternative stimuli was recorded. The positions of the stimuli were randomly rotated between test sheep to prevent stimulus location impacting on results.

#### Phase 2: Validating the Stimulus During the Attention Bias Test

Phase 2 of the pilot study aimed to validate whether the alternative stimulus selected during Phase 1 would be an appropriate replacement for food in the attention bias test. Observations during Phase 1 of the pilot study indicated the fiberglass model was preferred by test sheep, however, for easier replication in future studies, the side profile photo of an entire sheep (second preference) was selected for further use in Phase 2.

Ten sheep, different from those used in Phase 1, were tested individually in the attention bias test as described above to determine whether the chosen stimulus and its location were appropriate. The criteria for a successful stimulus were that (1) the test sheep showed interest in the stimulus, (2) test sheep did not appear to be fearful of the stimulus, and (3) test sheep were willing to move away from the stimulus during testing to explore the arena. To assess these criteria, the following behaviors were recorded during the 3 min test; latency to sniff the photo, time spent standing within 1 m of the photo, time spent looking at the photo, time spent looking at the dog window and number of grid sections entered (Figure 1). The group of sheep had been briefly habituated to the stimuli for 5 min prior to testing, to reduce the potential impact of stimulus novelty, which may induce fear (Boissy, 1995), on sheep responses during the test.

For the first three sheep that were tested, the front-on photo of a sheep face was also mounted next to the side profile photo as an additional attractant. The front-on photo was removed after three sheep were tested as it was deemed unnecessary. The other seven sheep were tested using the side-profile photo only.

Alternate locations were trialed to ensure sheep were attracted to the photo and not just the given location. The stimulus was initially placed in the location directly opposite the window (Location 3, Figure 1). After six sheep had been tested, the photo was moved to the wall next to the door (Location 1, Figure 1) and a further two sheep were tested. The photo was then moved to the opposite wall (Location 5, Figure 1) and the final two sheep were tested.

### Main Trial

#### Animal Details

Fifty non-lactating, non-pregnant Merino ewes (18–20 months old) with average bodyweight of 37.9 ± 2.8 kg were used in this experiment. Sheep had undergone routine handling prior to the current experiment and were therefore familiar with the presence of humans, but had no experience with the attention bias test or photos. Sheep had prior experience with dogs during routine on-farm management. The sheep were divided into two cohorts (*n* = 24 and 26) to be tested on separate days for logistical reasons. Treatment groups were evenly distributed between the two cohorts.

On day 0 of the main trial, all sheep were weighed and sorted into treatment groups balancing for bodyweight, then had numbers painted on their rumps for individual identification and were divided into two cohorts for testing. The first cohort then began pharmacological treatment the following afternoon (Day 1). The second cohort was returned to pasture and began treatment 2 days later so that attention bias testing would be staggered over 2 days. Treatment of the second cohort was identical to that described below.

#### Drug Details

The drug 4-chloro-DL-phenylalanine methyl ester hydrochloride (pCPA; Sigma-Aldrich, St. Louis, United States) was used to pharmacologically induce a state of depression. This drug has been shown to decrease brain serotonin levels and increase depression-like behaviors in a number of animal species including sheep (Kubala et al., 2008; Doyle et al., 2011; Stracke et al., 2017). The administration protocol for pCPA followed that described by Doyle et al. (2011), which caused a pessimistic judgment bias in sheep that was thought to reflect a depressed emotional state. Prior to administration, pCPA was dissolved in BP Water for Injection (Baxter, Toongabbie, Australia) at a rate of 50 mg/ml. pCPA was administered over five consecutive days at a rate of 40 mg/kg/day, administered in half doses each morning (8:00 AM) and afternoon (4:00 PM). The first dose was given on the afternoon of day 1 so that the final dose was given on the morning of day 6 prior to testing in the attention bias test. The solution was administered intraperitoneally (i.p.), by injection through the right paralumbar fossa, midway between the iliac crest and the last rib, approximately 50 mm below the end of the lumbar processes (Hurter, 1987).

The drug m-Chlorophenylpiperazine (mCPP; Tocris, Bristol, United Kingdom) was used to pharmacologically induce a state of anxiety. This drug has been shown to induce anxious states in humans and has been used a number of times to induce anxiety-like behaviors in sheep (Drake, 2006; Doyle et al., 2015; Lee et al., 2016; Monk et al., 2018). The administration protocol followed that described by Lee et al. (2016). Prior to treatment, mCPP was dissolved in BP Water for Injection at a rate of 86 mg/ml. mCPP was administered as a single intramuscular injection into the rump of the animal at a dose rate of 2 mg/kg, 30 min prior to testing in the attention bias test.

#### Drug Treatment Protocol

Animals were distributed between 3 treatment groups, “Anxious,” “Depressed,” and “Control” (*n* = 16 per group) balancing for bodyweight. All sheep received an injection of either their treatment group drug, or an equivalent volume of BP saline, for five consecutive days prior to testing, at 8 AM on the morning of testing and 30 min before undertaking the attention bias test as outlined in Table 1. In total, all sheep received 10 intraperitoneal injections and one intramuscular injection prior to undergoing the attention bias test. Two spare animals were included with cohort 2 and were treated in the same way as the Control group throughout the study. Due to the presence of abnormal stumbling behavior in some of mCPP treated sheep in mob 1, the spare sheep were also administered mCPP on the attention bias test day to ensure adequate numbers in the treatment group could be achieved if any animals had to later be removed from the study.

**Table 1:** Summary of drug treatments during the main trial.

Days	Time	Treatment group		
		Anxious (*n* = 18)	Depressed (*n* = 16)	Control (*n* = 16)
1	4 PM	Saline (i.p.)	pCPA (i.p.)	Saline (i.p.)
2–5	8 AM	Saline (i.p.)	pCPA (i.p.)	Saline (i.p.)
	4 PM	Saline (i.p.)	pCPA (i.p.)	Saline (i.p.)
5	4 PM	iButtons inserted and cohesive bandages applied to all animals		
6	8 AM	Saline (i.p.)	pCPA (i.p.)	Saline (i.p.)
	30 min prior to attention bias test	mCPP (i.m.)	Saline (i.m.)	Saline (i.m.)
	30 min post injection	Attention bias testing for all animals		

*The pharmacological agent and administration method are given for each treatment group at each time point. All sheep received a total of 10 intraperitoneal injections during days 1 to 6 and one intramuscular injection on day 6, 30 min prior to undergoing the attention bias test.*

Between intraperitoneal injections, all sheep were returned to paddocks adjacent to the handling facilities with access to pasture and fresh water. Sheep were mustered from the paddocks to the yards prior to each injection, then were monitored continuously for 30 min post injection before being returned to the paddocks.

On day 6, sheep were given their final intraperitoneal injection in the morning then were moved using a trailer to a second set of yards where the attention bias test was located. Sheep were left in the yards undisturbed for 2 h to allow them to settle following transportation. Individual sheep were given their single assigned intramuscular injection (Table 1) at 5 min intervals over a 2.5 h period so that each animal received their injection 30 min prior to undergoing the attention bias test. All injections and attention bias tests were completed within 3 h on test days.

#### Attention Bias Test

The main trial used the same attention bias test described for the pilot study, using a single photo of a sheep in side profile located on the wall directly opposite the dog window (Location 3, Figure 1). A high resolution copy of the photo is available in the Supplementary Figure 1. On day 6, the photo was placed in a holding yard with all sheep for a brief habituation period (5 min) approximately 30 min before beginning any injections.

#### Behavioral Measures in the Attention Bias Test

The behaviors recorded in the attention bias test are summarized in Table 2. Open and close mouthed vocalizations were scored on the day of testing by a hidden observer. The observer was positioned behind the opaque matting near the door wall of the test arena, out of view of the sheep. The same observer also recorded the dog’s posture, movements and vocalizations at the beginning of the test. Dog behavior was later categorized on a 3 point scale as: (1) quietly standing still, (2) lunging or crouching down, or (3) barked at the sheep with any posture. A score of 3 was given on 3 occasions. All other sheep behaviors in the attention bias test were collated from video footage using The Observer XT 12.0 (Noldus Information Technology, Wageningen, Netherlands). To determine whether treatment effects were evident for a shortened version of the test, separate analyses were also performed for the first 60 s of behaviors recorded during the test.

**Table 2:** Ethogram of behaviors recorded during the attention bias test.

Behavior	Definition
Attention	The direction in which the sheep is looking with binocular vision (Piggins and Phillips, 1996; Lee et al., 2016). The test arena was divided into six areas of attention: dog window, dog wall (including the window), photo, photo wall (including the photo), door wall, and back wall. Total duration of attention was recorded for each section, as well as latency to look at the photo.
Vigilance	Time spent with the head at or above shoulder height (Frid, 1997; Lee et al., 2016). Latency to become non-vigilant was also calculated.
Sniff photo	Number of times and latency to sniff the photo
Sniff environment	Number of times and latency to sniff the floor or walls of the test arena
Vocalizations	Number of open mouthed bleats and close mouthed bleats were recorded separately
Zones entered	Number of zones entered (1–9)
Zone duration	Total time spent in each of the 9 zones and latency to enter the zone closest to the dog
Elimination	Number of urinations or defecations

Sheep treated with mCPP were monitored for abnormal behaviors previously described by Doyle et al. (2015). These included ataxic gait, tail shaking, head shaking, body shaking, and head rolling. Abnormal behaviors were observed in 14 of the 18 mCPP treated sheep. Head shaking was observed in six sheep and tail shaking in nine sheep. No body shaking or head rolling was observed. Ataxic gait was observed in ive sheep, two of which stumbled multiple times while the other three stumbled only once.

#### Internal Body Temperature

Internal body temperature was recorded using Thermochron iButtons^®^ (Model number DS1922L-F5, accuracy 0.5°C, resolution 0.063°C, weight 3.3 g) (Embedded Data Systems, Lawrenceburg, United States) which were attached to blank (progesterone-free) Controlled Internal Drug Release devices (CIDR^®^, Zoetis, Melbourne, Australia) using polyolefin heat-shrink tubing, as described by Lea et al. (2008). A CIDR was inserted into the vagina of each sheep one day prior to testing using an applicator lubricated with obstetrical lubricant. The iButtons were set to log at an interval of 10 s beginning at 8:00 AM on the day of attention bias testing. Data were extracted using the program eTemperature version 8.32 (OnSolution, Castle Hill, Australia). The following time points were then selected for further analysis: -40 min, -30 (time of injection), -20, -10, 0 (start attention bias test), 3 (end of attention bias test), 8, 13 and 18 min. Data from two temperature loggers were missing due to technical faults and so post-attention bias test data for the last two animals tested each day were removed as these animals were handled soon after testing.

#### Data Loggers

HOBO^®^ Pendant G accelerometer data loggers were used to record steps during the attention bias test (dimensions: 58 mm × 33 mm × 23 mm, weight: 18 g) (Onset Computer Corporation, Pocasset, MA, United States). The HOBO^®^ Waterproof Shuttle and HOBOware^®^ Pro software (version 3.7.8) were used for programming and reading the HOBO loggers (Onset Computer Corporation, Pocasset, MA, United States). Data loggers were programmed to record at a logging interval of 0.03 s (33 Hz) when activated using a magnet [measurement range: ±3 gravitational force (*g*); accuracy: ±0.075 *g* at 25°C].

On the afternoon prior to testing, after receiving their injections, all sheep had a small silicone pad attached to the outside of their left hind leg in the middle of the cannon bone using veterinary cohesive bandage. The silicone pad had a small recess in which the HOBO loggers could be placed to keep them still and away from the leg. The following day, immediately prior to entering the attention bias test, a HOBO logger was activated using a magnet, then was tucked between the bandage and the silicone pad on the outside of the leg. All data loggers were removed at the end of the day after testing had been completed. Steps were calculated from the accelerometer data using an in-house program (Little, 2015).

#### Additional Measurements and Procedures

Blood samples were taken from all sheep on days 1 and 6 of the experiment for assessment of gene expression as part of another project (not further reported on here). Sample collection on day 6 occurred after all behavioral testing had been completed. The 2.5 ml blood samples were collected via jugular venipuncture using a 1′′ 18 g needle and PAXgene RNA protect vacutainers. On the morning of day 6 after sheep received their intraperitoneal injections, the rectal temperatures of the sheep were recorded to ensure no animals had developed a fever during the trial. No fevers were detected at this time.

### Statistical Methods

#### Pilot Study

Decisions in Phase 1 of the study were made based on the qualitative observations of two researchers at the time of the experiment. Summary statistics were obtained from the data using Microsoft Excel 2013.

#### Main Trial

Data were analyzed using R version 3.2.3 (R Core Team, 2015). *P*-values less than 0.05 were considered significant. All model residuals were checked for normality and homoscedasticity using Shapiro–Wilk test for normality and visual assessment of Q-Q and residuals vs. fitted values plots. Cohort (test day), test order and dog behavior score were fitted as fixed effects in all linear models and analyses of variance (ANOVA), however, none of these factors reached significance and were subsequently removed from all models using a backward elimination approach. *Post hoc* multiple comparisons were conducted using a Tukey’s method for adjustment of *P*-values.

Attention data were analyzed by one-way ANOVA. Data for attention toward the dog, photo and photo wall were log transformed to meet normality assumptions. Attention in the dog’s direction included a number of outliers with high leverage on the data, therefore these data were analyzed using an ANOVA on 10% trimmed means using the package WRS2 (Mair et al., 2017). The results from the ANOVA on trimmed means were also confirmed using Kruskal–Wallis non-parametric ANOVA. Vigilance duration data were analyzed by Kruskal–Wallis non-parametric ANOVA as the parametric model residuals did not meet normality assumptions and could not be improved by transformation (Grosjean and Ibanez, 2014). *Post hoc* multiple comparison tests for Kruskal–Wallis ANOVAs were performed using the package pgirmess (Giraudoux, 2016).

Time spent sniffing the photo and environment required transformation to meet normality assumptions, then were analyzed using one-way ANOVA. Time spent in the zone closest to the dog did meet normality assumptions and was analyzed using one-way ANOVA. Photo sniff frequency and number of zones entered were analyzed using generalized linear models with a Poisson distribution for count data. Number of steps and sniff environment frequency were analyzed using generalized linear models with a negative binomial distribution due to evidence of over-dispersion. Due to the low occurrence of vocalizations, vocalization data were analyzed using Fishers Exact Tests, examining the number of animals in each group which vocalized. Attention, vigilance, and exploratory behaviors during the first 60 s of testing were analyzed in the same way as for the full test duration.

All latency data were analyzed with Cox’s proportional hazards model using survival analysis, as described by Therneau and Grambsch (2000), Therneau, 2015, and Monk et al. (2018). These data included latencies to look at the photo, sniff the photo, sniff the environment, enter the zone closest to the dog and become non-vigilant. Animals which failed to perform each behavior within 180 s were deemed as censored results.

Body temperature data were analyzed using a maximum likelihood multilevel linear model to account for repeated measures on the same animals at each time point, fitting treatment, time and a treatment × time interaction as fixed effects (Field et al., 2012). Changes in temperature from the beginning of attention bias testing onward were then assessed in the same way. These data were obtained by subtracting the Time 0 (start of test) values from subsequent values for each animal.

## Results

### Pilot Study: Phase 1

The test sheep interacted with the fiberglass model more than any other stimulus in both a group setting and individually during the attention bias test (Table 3). The life-size photo of an entire sheep from the side received the second highest number of interactions (Table 3).

**Table 3:** The number of interactions a group of five sheep had with a variety of positive stimuli during Phase 1 of the pilot study.

Stimulus	Total number of interactions		Number of animals interacting	
	Group	Individual	Group	Individual
Fiberglass model	8	13	5	4
Body photo (side)	5	4	4	4
Body photo (front)	4	3	2	3
Face photo (front)	1	3	1	3
Face photo (side)	0	3	0	2

*The sheep (n = 5) were tested as a group in a small yard (Group) then individually in the attention bias test (Individual). Stimuli included a fiberglass model of a sheep and four life-size photos (Figure 2) of entire sheep or sheep faces.*

### Pilot Study: Phase 2

All sheep sniffed the stimulus during the test. Test sheep on average spent more than half their time standing within 1 m of the photo (Table 4). The two sheep which were slowest to sniff the photo (latencies of 60 and 63 s) spent the most time standing next to the photo (100 and 83% of time, respectively). All other sheep sniffed the photo within 30 s of beginning the test. The mean proportion of time spent standing near the photo was 57% when the photo was located directly opposite the dog window (*n* = 6) and 58% when the photo was moved to one of the other locations (*n* = 4). The mean, minimum, and maximum latencies, frequencies and durations of all sheep behavior in the test are given in Table 4.

**Table 4:** Mean, minimum, and maximum values for behaviors of sheep during Phase 2 of the pilot study, during the attention bias test.

Statistic	Sniff photo latency (s)	Sniff photo frequency	Standing near photo (%)	Looking at photo (%)	Looking at window (%)	Zones entered
Mean	18.9	3.4	57.4	21.3	15.8	5.0
Minimum	0	1	23	3	8	1
Maximum	63	7	100	41	30	9

### Main Trial

#### Attention Behaviors

**FIGURE 3:**
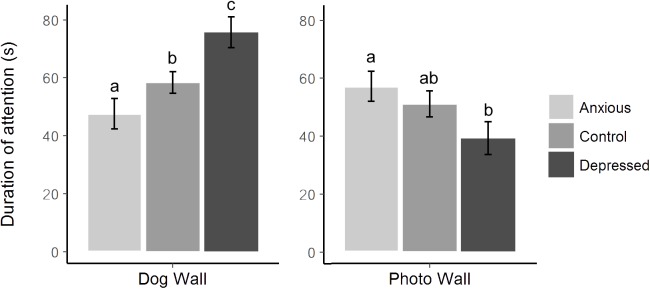
Mean ± SEM time spent looking toward the dog wall versus the photo wall for each treatment group during attention bias testing. Different letters represent significant differences between treatment groups within each section of the graph as determined using *post hoc* analyses.

**FIGURE 4:**
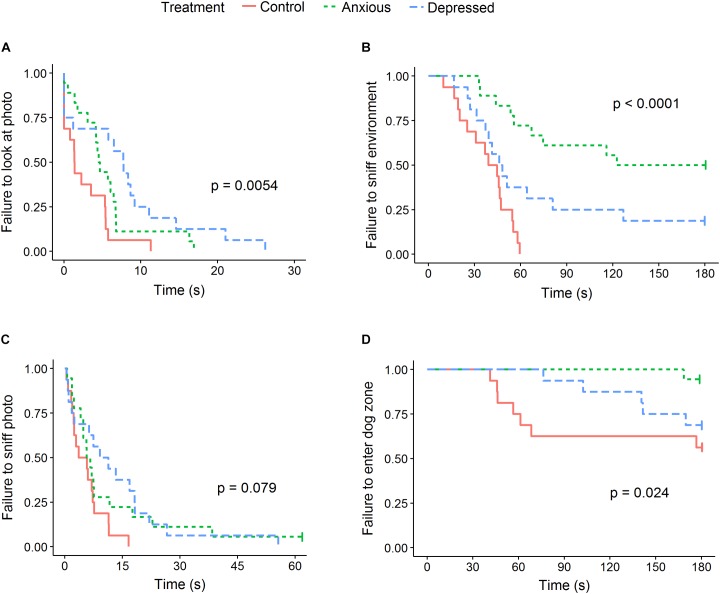
Kaplan–Meier curves for latency to look at the photo **(A)**, sniff the environment **(B)**, sniff the photo **(C)**, and enter the zone closest to the dog **(D)**. Each time an animal exhibited the given behavior, the probability on the *Y*-axis drops. Latencies to look at and sniff the photo are given for the first 60 s of testing only as no further events occurred after this time. The censored result in the anxious group for latency to sniff the photo remained censored for the duration of the 180 s test.

No differences were seen between treatment groups for time spent looking directly at the closed dog window or photo, however, differences were seen between groups in time spent looking at the dog and photo walls (Table 5 and Figure 3). Specifically, Depressed sheep spent the most time looking toward the dog wall and least time looking toward the photo wall, while Anxious sheep spent the most time looking toward the photo wall and least time looking toward the dog wall (Figure 3). Both Anxious and Depressed sheep were slower to look at and sniff the photo than Control animals, although the difference between the Control and Anxious groups only tended toward significance (Table 6 and Figure 4). There were no differences between groups for duration of attention toward the door wall or back wall.

**Table 5:** Mean ± SEM behavioral responses of sheep in each treatment group during the attention bias test.

Behavioral measure	Control	Anxious	Depressed	Test value	*P*-value
Attention to dog window (s)	22.6 ± 2.4	20.7 ± 2.7	27.3 ± 3.0	*F*_(2,47)_ = 2.44	0.099
Attention to dog wall (s)	58.4 ± 3.8^a^	47.6 ± 5.3^b^	75.8 ± 5.3^c^	*F*_(2,47)_ = 7.09	<0.001
Attention to photo (s)	37.3 ± 4.4	36.7 ± 3.9	29.1 ± 4.6	*F*_(2,47)_ = 1.57	0.22
Attention to photo wall (s)	51.0 ± 5.2^ab^	57.1 ± 4.9^a^	39.2 ± 5.2^b^	*F*_(2,47)_ = 4.96	0.011
Attention to door wall (s)	45.5 ± 4.0	49.4 ± 3.8	41.4 ± 4.0	*F*_(2,47)_ = 1.03	0.37
Attention to back wall (s) ^1^	3.1 ± 0.2 (22.3)	3.0 ± 0.2 (19.8)	3.0 ± 0.2 (19.3)	*F*_(2,47)_ = 0.19	0.83
Vigilance (mean rank duration) ^2^	14.5 ± 2.3^a^ (160)	36 ± 3.3^b^ (171)	30.5 ± 3.7^b^ (168)	*X*^2^_(2)_ = 12	0.002
Standing near photo (s)	121 ± 9.9^a^	153 ± 9.3^b^	108 ± 9.9^a^	*F*_(2,47)_ = 5.91	0.005
Sniff photo (*n*) ^1^	1.9 ± 0.1^a^ (6.6)	1.4 ± 0.1^b^ (1.4)	1.5 ± 0.1^b^ (1.5)	*X*^2^_(2)_ = 11.4	0.003
Sniff environment (*n*) ^1^	1.9 ± 0.2*^a^* (6.7)	0.8 ± 0.2^b^ (2.2)	1.4 ± 0.2^ab^ (4.1)	*X*^2^_(2)_ = 11.7	0.003
Zones entered (*n*) ^1^	1.74 ± 0.1^a^ (5.7)	0.94 ± 0.2^b^ (2.6)	1.68 ± 0.1^a^ (5.4)	*X*^2^_(2)_ = 25.1	<0.001

*^a,b,c^Different superscripts within rows indicate a significant difference between treatments as determined using post hoc analyses. ^1^Least squares means are given on the log scale, back-transformed means are given in parentheses. ^2^Mean ranks are given, raw means are given in parentheses.*

**Table 6:** Hazard ratios for latency to look at the photo, sniff the photo, sniff the environment, and enter the zone closest to the dog window as affected by treatment group.

Latency to	Group	Mean^1^	Coefficient^2^	SE (coeff)	Hazard ratio^3^	Wald (z)	*P*
Look at photo	**Control**	2.7	**Reference**				
	Anxious	5.6	−0.674	0.35	0.51 (0.25–1.02)	−1.91	0.057
	Depressed	8.0	−1.203	0.39	0.30 (0.14–0.65)	−3.08	**0.002**
	**Anxious**		**Reference**				
	Depressed		−0.529	0.37	0.59 (0.29–1.20)	−1.45	0.147
Sniff photo	**Control**	5.6	**Reference**				
	Anxious	18.4	−0.644	0.37	0.53 (0.26–1.08)	−1.75	0.08
	Depressed	13.3	−0.787	0.38	0.46 (0.22–0.95)	−2.09	**0.037**
	**Anxious**		**Reference**				
	Depressed		−0.143	0.35	0.87 (0.44–1.70)	−0.41	0.69
Sniff environment	**Control**	38.1	**Reference**				
	Anxious	123.3	−1.892	0.46	0.15 (0.06–0.37)	−4.12	**<0.001**
	Depressed	73.5	−0.894	0.40	0.41 (0.19–0.90)	−2.23	**0.026**
	**Anxious**		**Reference**				
	Depressed		0.998	0.44	2.7 (1.2–6.4)	2.29	**0.022**
Enter zone closest to dog	**Control**	132.0	**Reference**				
	Anxious	179.4	−2.395	1.07	0.09 (0.01–0.74)	−2.24	**0.025**
	Depressed	163.2	−0.5684	0.59	0.57 (0.18–1.79)	−0.97	0.333
	**Anxious**		**Reference**				
	Depressed		1.83	1.10	6.20 (0.73–53.0)	1.67	0.095

*^1^Raw mean latencies are given. ^2^Regression coefficient from the Cox-proportional hazards model. ^3^95% confidence interval given in parentheses. Significant P-values are shown in bold.*

#### Vigilance and Other Behaviors Expressed During Testing

Anxious and Depressed groups were more vigilant than Control animals during the attention bias test (Table 5). The Anxious and Depressed groups did not differ. Latency to become non-vigilant only tended to differ between groups [*X*^2^ (2) = 5.39, *P* = 0.068].

Sheep in the Anxious and Depressed groups were less likely to sniff the photo than Control animals, but did not differ from one another (Tables 5, 6). Anxious sheep were less likely to sniff the environment, spent more time standing near the photo, entered fewer zones and were less likely to enter the zone closest to the dog than Control animals, while the Depressed group did not differ from Control animals for these behaviors (Figure 4 and Tables 5, 6). The sniff environment frequency of the Depressed group was intermediate between the Control and Anxious groups, but did not significantly differ from either treatment, while latency to sniff the environment differed significantly between all groups (Figure 4 and Tables 5, 6). Number of steps taken did not differ between treatment groups (40.5, 30.1, and 37.4 steps for Control, Anxious, and Depressed groups, respectively, *P* = 0.33).

More sheep in the Control group vocalized than sheep in the Anxious and Depressed groups. This was consistent for both open mouthed vocalizations (8, 2, and 0 sheep in the Control, Anxious, and Depressed groups, respectively, *P* < 0.001) and close mouthed vocalizations (12, 6, and 7 sheep, respectively, *P* = 0.05). Five sheep urinated during the attention bias test, all of which were in the Anxious group. No animals defecated during testing. Only two sheep approached and sniffed the closed dog window, both of which were in the Control group.

### Body Temperature

**FIGURE 5:**
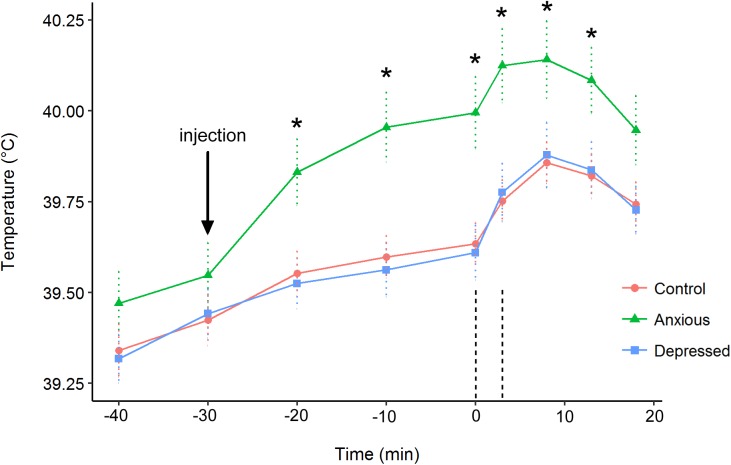
Mean (±SE) body temperatures for the Control (●), Depressed (■), and Anxious (▲) groups. The arrow denotes the time of injection prior to testing. The vertical dashed lines indicate the beginning and end of attention bias testing. The “^∗^” symbol denotes a significant difference between the Anxious group mean and the other groups as determined using a repeated measures linear mixed model.

In the repeated measures analysis on body temperature data, the Treatment × Time interaction was significant [*X*^2^(16) = 50.8, *P* < 0.001]. The main effects were also significant for both treatment [*X*^2^(2) = 9.8, *P* = 0.008] and time [*X*^2^(8) = 900.2, *P* < 0.001]. Contrasts indicated that body temperature did not differ between groups at times -40 and -30 (*P* > 0.1). At 15 min post attention bias testing, the Anxious group had a significantly higher body temperature than the Depressed group [*t*(45) = -2.1, *P* = 0.044], but only tended to be higher than the Control group [*t*(45) = -1.9, *P* = 0.069]. The Anxious group had a higher body temperature than the Control and Depressed groups at all other time points (Figure 5). The Control and Depressed groups did not differ at any time point.

**FIGURE 6:**
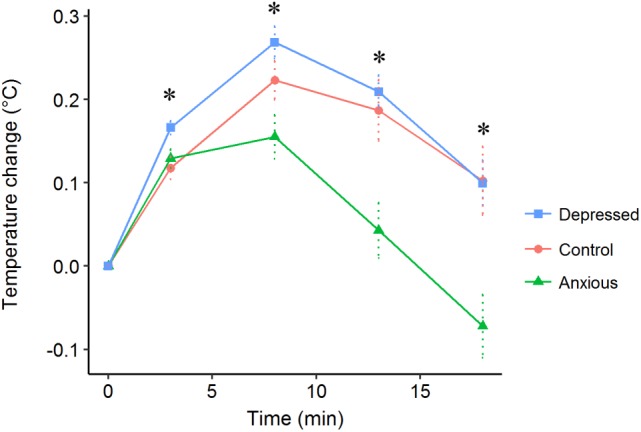
Mean (±SE) change in body temperatures after beginning attention bias testing (Time 0) for the Control (●), Depressed (■), and Anxious (▲) groups. Sheep completed the attention bias test after 3 min. The “^∗^” symbol denotes a significant difference between treatment groups as determined using a repeated measures linear mixed model and *post hoc* tests.

The Treatment × Time interaction was significant for change in body temperature after attention bias testing [*X*^2^(8) = 57.9, *P* < 0.001]. The Depressed group showed a greater increase in body temperature than the other two groups immediately after attention bias testing (Time 3 min, LS mean 0.17°C), while the Control and Anxious groups did not differ (LS means 0.12 and 0.13°C, respectively, *P* = 0.008) (Figure 6). At all other time points, the Anxious group differed from the Control and Depressed groups (*P* < 0.05) while the Control and Depressed groups did not differ (*P* > 0.05).

#### Behavior in the First 60 s

No differences were found between the Control group and other treatments for any attention behaviors in the first 60 s of testing (*P* > 0.05), however, the Depressed group spent more time looking toward the dog wall than the Anxious group (Table 7). Results for latency to look at and sniff the photo did not differ from the full length test as no further events occurred after 60 s for these data. No differences were seen between groups for sniff photo frequency (Table 7).

**Table 7:** Mean ± SEM behavioral responses of sheep during the first 60 s of the attention bias test.

Behavioral measure	Control	Anxious	Depressed	Test value	*P*-value
Attention to dog window (s)	9.4 ± 1.0	9.1 ± 1.5	9.7 ± 1.1	*F*_(2,47)_ = 0.6	0.58
Attention to dog wall (s)	9.4 ± 1.3^ab^	7.1 ± 1.3^a^	13.0 ± 1.3^b^	*F*_(2,47)_ = 5.23	0.008
Attention to photo (s)	15.3 ± 1.5	15.4 ± 1.4	11.3 ± 1.5	*F*_(2,47)_ = 2.54	0.089
Attention to photo wall (s)	19.4 ± 1.8	20.2 ± 1.7	15.7 ± 1.8	*F*_(2,47)_ = 1.83	0.17
Vigilance (mean rank duration) ^1^	13.9 ± 2.4^a^ (54.9)	27.8 ± 3.3^b^ (57.8)	34.6 ± 2.8^b^ (58.9)	*X*^2^_(2)_ = 17	<0.001
Standing near photo (s)	23.8 ± 3.6^ab^ (48.1)	34.7 ± 2.7^a^ (55.6)	16.8 ± 3.1^b^ (41.3)	*X*^2^_(2)_ = 13	0.001
Sniff photo (*n*) ^2^	1.3 ± 0.1 (3.8)	1.0 ± 0.1 (2.8)	1.0 ± 0.2 (2.8)	*X*^2^_(2)_ = 2.9	0.23
Sniff environment (*n*) ^2,3^	0.7 ± 0.2 (2.1) ^a^	−1.3 ± 0.5 (0.3) ^b^	−0.3 ± 0.3 (0.8) ^a^	*X*^2^_(2)_ = 28.1	<0.001
Zones entered (*n*) ^2^	1.25 ± 0.1^a^ (3.5)	0.61 ± 0.2^b^ (1.8)	1.32 ± 0.1^a^ (3.8)	*X*^2^_(2)_ = 13.5	<0.001

*^a,b^Different superscripts within rows indicate a significant difference between treatments as determined using post hoc analyses. ^1^Mean ranks are given, raw means are given in parentheses. ^2^Least squares means are given on the log scale, back-transformed means are given in parentheses. ^3^Generalized linear model fitting a Poisson distribution can generate negative least squares means on the log scale.*

Differences between treatment groups in the first 60 s of testing were consistent with the full length test for vigilance duration, time standing near the photo, sniff environment frequency and number of zones entered (Table 7). Latency to become non-vigilant significantly differed between treatment groups (likelihood ratio = 6.91, *df* = 2, *P* = 0.032), with Depressed sheep taking significantly longer to become non-vigilant than Control animals (mean latencies 48 and 35 s, respectively, *z* = -2.51, *P* = 0.012). The Anxious group did not differ from the Control or Depressed groups. Latency to sniff the environment differed significantly between all groups (likelihood ratio = 18.6, *df* = 2, *P* < 0.001).

## Discussion

Sheep in an induced anxious state displayed an attention bias toward the positive stimulus and away from the threat compared to control animals, which contrasts with our hypothesis and previous studies (Lee et al., 2016; Monk et al., 2018). We suggest the differences in animal responses between the current and previous methods are likely due to the social aspect of the new positive stimulus. The threat provided by the test procedure is acutely stressful, as indicated by the SIH seen in all treatment groups. Acute stress responses involve allocation of resources away from non-essential functions, such as feeding behavior, toward biological functions that aid survival or escape (Sherwood et al., 2005), which for a gregarious species with a strong flocking instinct can involve locating and staying with conspecifics (Lynch et al., 1992). Thus, attention toward conspecifics may be an appropriate response for sheep in a threatening situation, and be enhanced by anxiety. Modification of the test to replace feed with a photo changed the direction of the attention bias in anxious animals, indicating a context specific interpretation of responses is required for different attention bias test designs.

Depressed sheep showed greater attention toward the threat than Control animals as predicted, indicating the modified attention bias test may be used to assess states of depression in sheep. This result supports our hypothesis of an attention bias toward threat and/or away from positive stimuli in depressed individuals. Interestingly, the Depressed sheep showed an opposite bias in attention to that seen in Anxious sheep. If attention to conspecifics is an effective coping strategy when faced with a threat, as suggested above, then increased attention to threat in the Depressed group may represent an inappropriate coping response. This interpretation is supported by evidence that humans with depression are more likely to display maladaptive coping strategies to stressful situations than healthy individuals (Billings and Moos, 1984; Nolen-Hoeksema et al., 1999). On the other hand, enhanced attention to the threat may suggest the Depressed animals were less anxious during the test than Control animals. This interpretation is not supported by changes in body temperature (SIH) following testing, where the Depressed group exhibited a small, but significantly greater increase in body temperature, suggesting they were more stressed by the testing procedure than the Control sheep (Bouwknecht et al., 2007). Alternatively, or perhaps additionally, reduced attention toward the positive stimuli might reflect symptoms of anhedonia or social withdrawal which are observed in humans with depression (Guze, 1995) and in depression-like animal models (Moreau et al., 1992; Barr and Phillips, 1999). In any case, sheep in both Anxious and Depressed states displayed attention biases which significantly differed from the Control group. The bidirectional nature of the attention bias between the different types of negative states could potentially make interpretation of animal responses difficult during future application of the test. Consideration of other behaviors during the test may help to differentiate the affective states of the animals.

Amongst other behaviors expressed during the test, both Anxious and Depressed sheep appeared to show signs of an increased fear response compared to the Control group. Vigilance behavior, typically associated with fearful and anxious states (Wemelsfelder and Farish, 2004), was higher in the Anxious and Depressed groups. SIH and an increased urination frequency observed in the Anxious group are signs of autonomic hyperarousal, consistent with an anxious state and the findings of previous studies (Sherwood et al., 2005; Erhard et al., 2006; Pedernera-Romano et al., 2010; Lee et al., 2017). A greater increase in body temperature during testing in the Depressed group suggests an increased stress response to the test itself. Anxious and Depressed groups vocalized less than control animals, which may indicate fearfulness in contexts where a potential predator is present (Romeyer and Bouissou, 1992; Beausoleil et al., 2005). Anxious sheep were also least likely to sniff the environment and photograph, again suggesting that they were more fearful (Romeyer and Bouissou, 1992; Beausoleil et al., 2005, 2012). No differences were seen between any of the groups for number of steps taken, which is consistent with previous studies in sheep (Doyle et al., 2011; Lee et al., 2016; Monk et al., 2018). Locomotion behaviors can be context specific and difficult to interpret due to an inability to distinguish between conflicting motivations such as exploratory or fleeing behaviors (Romeyer and Bouissou, 1992). As such, the sniffing behaviors may better reflect exploration or interest in the stimuli and environment during the attention bias test. Overall, both Anxious and Depressed animals showed signs of increased fear during the test compared to control animals. In the context of the modified attention bias test, key fear related responses such as vigilance and exploratory behavior may be used to determine when an animal is in a more negative affective state, then the attention behaviors may be used to differentiate between states of depression and anxiety. Considering these behaviors together, as well as the context in which the test is applied, will allow for a greater understanding of the affective states of animals using the modified attention bias test.

The alternative positive stimulus appears to have been a successful replacement for food in the attention bias test, allowing for a more standardized application of the test and clearer interpretation of results when using pharmacological treatments. All but one animal had sniffed the photograph within the first 60 s of the attention bias test, indicating an absence of fear toward this stimulus (Romeyer and Bouissou, 1992; Beausoleil et al., 2005, 2012). Animals also spent a high proportion of their time in the test standing next to the photograph, suggesting the photograph was perceived as positive by the test animals and may have been recognized as a conspecific. The modified method removed variation from appetite and feeding motivation from the test, however, it also potentially added variation from social motivation to the test. We suggest the former is likely to have a greater impact on results, with variation occurring not only between animals, but also within animals over time as they spend more time without feed prior to testing. As such, we suggest the modified method is an improvement over the original method, however, further studies to directly compare the test designs would be useful to confirm this suggestion. Further, in the original method, sheep could position themselves in the arena so that they could look toward the dog window while eating food, making it difficult to differentiate attention to the positive versus negative stimuli. In the modified method, attention to the threat and attention to the positive stimulus are mutually exclusive, allowing for clearer interpretation of behavior. If future studies choose to use food as a positive stimulus, we suggest the food should be positioned in a way that sheep cannot easily look at the threat while eating, to more clearly differentiate their allocation of attention between these stimuli.

When interpreting the behavioral responses observed in the test, it is important to consider some of the other factors which may have impacted on animal behavior. The test procedure itself may induce some level of anxiety, however, exposure to the test as an environmental source of anxiety was consistent across all treatment groups in the current study. For simplicity, we have assumed that an animal’s displayed response is most strongly determined by the affective state it brings to the test arena, rather than by a new affective state arising from exposure to environmental stimuli during the course of the test. This assumption is supported by the treatment differences observed in both the current and previous studies, between groups with differing initial affective states (Lee et al., 2016; Monk et al., 2018). Whether the initial affective state of the animal further influences its behavioral response to anxiety-inducing components of the test procedure remains unknown.

Another factor which may have influenced behavioral responses during the test is the use of pharmacological treatments. Some of the animals in the Anxious group showed some form of abnormal behaviors in response to the drug treatment. Abnormal behaviors have been observed previously in young sheep administered mCPP at a dose rate of 2 mg/kg (Doyle et al., 2015; Monk et al., 2018) but had not previously been observed in adult sheep using the same dose rate (Lee et al., 2009, 2016). In the current study, most of the behaviors observed were tail or head shakes, which we expect did not impact on other behaviors in the test. However, a few sheep showed signs of ataxic gait, where it appeared they failed to lift their rear foot enough when stepping and therefore stumbled. If some of the sheep were having difficulty walking, this may have reduced behaviors related to locomotion such as number of steps and escape attempts. During visual inspection of the data, stumbling behaviors did not appear to have a pronounced impact on number of steps taken during the test, with no mCPP treated sheep appearing as outliers within the stepping data. Nevertheless, it cannot be ruled out that locomotion was impacted by the drug. Although the abnormal behaviors observed in this study were mild, it is suggested further studies using mCPP in sheep should use a lower dose rate of 1 to 1.5 mg/kg, as suggested by Doyle et al. (2015), regardless of animal age.

Further validation and refinement of the modified methodology will help to improve the application of the attention bias test in future studies and increase our understanding of animal responses during testing. The current study demonstrated that states of depression and anxiety can influence responses during the test, however, the effects of other types of affective states on test performance need to be ascertained through further study. The influence of positive affective states could be of particular interest, as the presence of positive affective states make up an important component of animal well-being but have so far been relatively understudied when compared to negative states (de Vere and Kuczaj, 2016). A guiding principle for additional refinements to the test method should be to ensure its utility for application on farms. Monk et al. (2018) suggested that the original test duration could be shortened to enable its application to larger numbers of animals. However, the results of the current study suggest this would not be appropriate for the modified attention bias test method, as attention behaviors did not differ between groups during the first 60 s of testing. Automation of behavioral measurement during the test may facilitate collation of behavioral data, which can be a lengthy and labor intensive process. Finally, any modifications of the test arena design should ensure the test is suitable for use in existing handling facilities in on-farm settings.

## Conclusion

The modified attention bias test presented in the current study potentially offers a method for assessing different types of negative affective states in sheep. Further, by assessing the direction in which an attention bias occurs, toward or away from the threatening stimulus, we may be able to differentiate between distinct anxiety and depression-like states. Modification of the method to replace the food with a photograph of a conspecific allows for more standardized application of the test and eliminates variation in results caused by changes in feeding motivation. Overall, these results support the use of the modified attention bias test for further research of affective states in sheep and potentially other livestock.

## Data Availability

The dataset generated for this study can be found in the CSIRO data access portal (DAP) (https://doi.org/10.25919/5b5016c4bca25).

## Author Contributions

CL attained the funding for the project. CL and JM contributed to the conception and design of the study. JM and SB performed the experiments. JM performed the statistical analysis and wrote the first draft of the manuscript. CL, IC, and SB assisted with interpretation of results and revision of the manuscript. All authors read and approved the submitted version of the manuscript.

## Conflict of Interest Statement

The authors declare that the research was conducted in the absence of any commercial or financial relationships that could be construed as a potential conflict of interest.

## Supplementary Material

FIGURE S1 Photograph of a sheep in side-profile. The photograph was printed to be approximately life-size on A0, matte, 200 gsm cardstock then was cut out and mounted to 5 mm thick black corflute board using spray adhesive for use in the modified attention bias test.

## References

[B1] Armstrong T. Olatunji B. O. (2012). Eye tracking of attention in the affective disorders: a meta-analytic review and synthesis. *Clin. Psychol. Rev.* 32 704–723. 10.1016/j.cpr.2012.09.004 23059623 PMC3556338

[B2] Bar-Haim Y. Lamy D. Pergamin L. Bakermans-Kranenburg M. J. van IJzendoorn M. H. (2007). Threat-related attentional bias in anxious and nonanxious individuals: a meta-analytic study. *Psychol. Bull.* 133 1–24. 10.1037/0033-2909.133.1.1 17201568

[B3] Barr A. M. Phillips A. G. (1999). Withdrawal following repeated exposure to d-amphetamine decreases responding for a sucrose solution as measured by a progressive ratio schedule of reinforcement. *Psychopharmacology* 141 99–106. 10.1007/s002130050812 9952071

[B4] Beausoleil N. J. Blache D. Stafford K. J. Mellor D. J. Noble A. D. L. (2012). Selection for temperament in sheep: domain-general and context-specific traits. *Appl. Anim. Behav. Sci.* 139 74–85. 10.1016/j.applanim.2012.02.020

[B5] Beausoleil N. J. Stafford K. J. Mellor D. J. (2005). Sheep show more aversion to a dog than to a human in an arena test. *Appl. Anim. Behav. Sci.* 91 219–232. 10.1016/j.applanim.2004.10.008

[B6] Billings A. G. Moos R. H. (1984). Coping, stress, and social resources among adults with unipolar depression. *J. Pers. Soc. Psychol.* 46 877–891. 10.1037/0022-3514.46.4.877 6737198

[B7] Boissy A. (1995). Fear and fearfulness in animals. *Q. Rev. Biol.* 70 165–191. 10.1086/516403 7610234

[B8] Bouissou M. F. Porter R. H. Boyle L. Ferreira G. (1996). Influence of a conspecific image of own vs. different breed on fear reactions of ewes. *Behav. Process.* 38 37–44. 10.1016/0376-6357(96)00016-2 24897628

[B9] Bouwknecht J. A. Olivier B. Paylor R. E. (2007). The stress-induced hyperthermia paradigm as a physiological animal model for anxiety: a review of pharmacological and genetic studies in the mouse. *Neurosci. Biobehav. Rev.* 31 41–59. 10.1016/j.neubiorev.2006.02.002 16618509

[B10] Bradley B. P. Mogg K. Lee S. C. (1997). Attentional biases for negative information in induced and naturally occurring dysphoria. *Behav. Res. Ther.* 35 911–927. 10.1016/S0005-7967(97)00053-3 9401132

[B11] Bradley B. P. Mogg K. Millar N. White J. (1995). Selective processing of negative information: effects of clinical anxiety, concurrent depression, and awareness. *J. Abnorm. Psychol.* 104 532–536. 10.1037//0021-843x.104.3.532 7673577

[B12] de Vere A. J. Kuczaj S. A. (2016). Where are we in the study of animal emotions? *Wiley Interdiscip. Rev. Cogn. Sci.* 7 354–362. 10.1002/wcs.1399 27327075

[B13] Dodd C. L. Pitchford W. S. Hocking Edwards J. E. Hazel S. J. (2012). Measures of behavioural reactivity and their relationships with production traits in sheep: a review. *Appl. Anim. Behav. Sci.* 140 1–15. 10.1016/j.applanim.2012.03.018

[B14] Doyle R. E. Hinch G. N. Fisher A. D. Boissy A. Henshall J. M. Lee C. (2011). Administration of serotonin inhibitor p-Chlorophenylalanine induces pessimistic-like judgement bias in sheep. *Psychoneuroendocrinology* 36 279–288. 10.1016/j.psyneuen.2010.07.018 20833479

[B15] Doyle R. E. Lee C. McGill D. M. Mendl M. (2015). Evaluating pharmacological models of high and low anxiety in sheep. *PeerJ* 3:e1510. 10.7717/peerj.1510 26713255 PMC4690367

[B16] Drake K. A. (2006). *The Neurophysiological Regulation of Temperament in Sheep.* Doctoral thesis, University of New England, Armidale.

[B17] Erhard H. W. Elston D. A. Davidson G. C. (2006). Habituation and extinction in an approach–avoidance test: an example with sheep. *Appl. Anim. Behav. Sci.* 99 132–144. 10.1016/j.applanim.2005.10.008

[B18] Field A. P. Miles J. Field Z. (2012). *Discovering Statistics using R.* London: SAGE.

[B19] Forkman B. Boissy A. Meunier-Salaün M. C. Canali E. Jones R. B. (2007). A critical review of fear tests used on cattle, pigs, sheep, poultry and horses. *Physiol. Behav.* 92 340–374. 10.1016/j.physbeh.2007.03.016 18046784

[B20] Frid A. (1997). Vigilance by female Dall’s sheep: interactions between predation risk factors. *Anim. Behav.* 53 799–808. 10.1006/anbe.1996.0345

[B21] Giraudoux P. (2016). *pgirmess: Data Analysis in Ecology. R Package Version 1.6.4.* Available at: https://cran.r-project.org/package=pgirmess.

[B22] Grosjean P. Ibanez F. (2014). *pastecs: Package for Analysis of Space-Time Ecological Series. R package version 1.3-18.* Available at: https://cran.r-project.org/package=pastecs.

[B23] Guze S. B. (1995). *Diagnostic and Statistical Manual of Mental Disorders*, 4th Edn Berlin: Springer, 10.1176/ajp.152.8.1228

[B24] Harrison A. A. Liem Y. T. B. Markou A. (2001). Fluoxetine combined with a serotonin-1A receptor antagonist reversed reward deficits observed during nicotine and amphetamine withdrawal in rats. *Neuropsychopharmacology* 25 55–71. 10.1016/S0893-133X(00)00237-2 11377919

[B25] Hurter L. R. (1987). A preliminary investigation into the efficacy of intraperitoneal vaccination of sheep and goat kids against heartwater. *Onderstepoort J. Vet. Res.* 54 507–508. 3448579

[B26] Kubala K. H. McGinnis M. Y. Anderson G. M. Lumia A. R. (2008). The effects of an anabolic androgenic steroid and low serotonin on social and non-social behaviors in male rats. *Brain Res.* 1232 21–29. 10.1016/j.brainres.2008.07.065 18692488

[B27] Lea J. M. Niemeyer D. D. O. Reed M. T. Fisher A. D. Ferguson D. M. (2008). Development and validation of a simple technique for logging body temperature in free-ranging cattle. *Aust. J. Exp. Agric.* 48 741–745. 10.1071/EA07422

[B28] Lee C. Cafe L. M. Robinson S. L. Doyle R. E. Lea J. M. Small A. H. (2017). Anxiety influences attention bias but not flight speed and crush score in beef cattle. *Appl. Anim. Behav. Sci.* 205 210–215. 10.1016/j.applanim.2017.11.003

[B29] Lee C. Reed M. T. Gresset F. Doyle R. E. Fisher A. D. (2009). “The effect of anxiety on memory and learning performance in sheep,” in *Proceedings of the International Society of Applied Ethology Congress*, Cairns.

[B30] Lee C. Verbeek E. Doyle R. Bateson M. (2016). Attention bias to threat indicates anxiety differences in sheep. *Biol. Lett.* 12:20150977. 10.1098/rsbl.2015.0977 27277950 PMC4938034

[B31] Little B. (2015). *CSIRO Livestock Phenomics Information System (version 1.0).*

[B32] Lynch J. J. Hinch G. N. Adams D. B. (1992). *The Behaviour of Sheep: Biological Principles and Implications for Production.* Victoria: CSIRO.

[B33] Mair P. Schoenbrodt F. Wilcox R. (2017). *WRS2: Wilcox Robust Estimation and Testing.* Avaiable at: https://cran.r-project.org/web/packages/WRS2/citation.html.

[B34] Mathews A. Ridgeway V. Williamson D. A. (1996). Evidence for attention to threatening stimuli in depression. *Behav. Res. Ther.* 34 695–705. 10.1016/0005-7967(96)00046-0 8936752

[B35] Mogg K. Bradley B. P. Williams R. (1995). Attentional bias in anxiety and depression: the role of awareness. *Br. J. Clin. Psychol.* 34 17–36. 10.1111/j.2044-8260.1995.tb01434.x 7757037

[B36] Monk J. E. Doyle R. E. Colditz I. G. Belson S. Cronin G. M. Lee C. (2018). Towards a more practical attention bias test to assess affective state in sheep. *PLoS One* 13:e0190404. 10.1371/journal.pone.0190404 29293636 PMC5749786

[B37] Moreau J.-L. Jenck F. Martin J. R. Mortas P. Haefely W. E. (1992). Antidepressant treatment prevents chronic unpredictable mild stress-induced anhedonia as assessed by ventral tegmentum self-stimulation behavior in rats. *Eur. Neuropsychopharmacol.* 2 43–49. 10.1016/0924-977X(92)90035-7 1638173

[B38] Nolen-Hoeksema S. Larson J. Grayson C. (1999). Explaining the gender difference in depressive symptoms. *J. Pers. Soc. Psychol.* 77 1061–1072. 10.1037/0022-3514.77.5.1061 10573880

[B39] Paul E. S. Harding E. J. Mendl M. (2005). Measuring emotional processes in animals: the utility of a cognitive approach. *Neurosci. Biobehav. Rev.* 29 469–491. 10.1016/j.neubiorev.2005.01.002 15820551

[B40] Peckham A. D. McHugh R. K. Otto M. W. (2010). A meta-analysis of the magnitude of biased attention in depression. *Depress. Anxiety* 27 1135–1142. 10.1002/da.20755 21049527

[B41] Pedernera-Romano C. Ruiz de la Torre J. L. Badiella L. Manteca X. (2010). Effect of perphenazine enanthate on open-field test behaviour and stress-induced hyperthermia in domestic sheep. *Pharmacol. Biochem. Behav.* 94 329–332. 10.1016/j.pbb.2009.09.013 19799930

[B42] Piggins D. Phillips C. J. C. (1996). The eye of the domesticated sheep with implications for vision. *Anim. Sci.* 62 301–308. 10.1017/S1357729800014612

[B43] R Core Team (2015). *R: A Language and Environment for Statistical Computing.* Available at: https://www.r-project.org/

[B44] Romeyer A. Bouissou M.-F. (1992). Assessment of fear reactions in domestic sheep, and influence of breed and rearing conditions. *Appl. Anim. Behav. Sci.* 34 93–119. 10.1016/S0168-1591(05)80060-7

[B45] Sherwood L. Klandorf H. Yancey P. H. (2005). *Animal Physiology: From Genes to Organisms.* Forest Lodge Rd, CA: Thomson/Brooks/Cole.

[B46] Stracke J. Otten W. Tuchscherer A. Puppe B. Düpjan S. (2017). Serotonin depletion induces pessimistic-like behavior in a cognitive bias paradigm in pigs. *Physiol. Behav.* 174 18–26. 10.1016/j.physbeh.2017.02.036 28257935

[B47] Therneau T. (2015). *A Package for Survival Analysis in S. version 2.38.* Available at: http://cran.r-project.org/package=survival

[B48] Therneau T. M. Grambsch P. M. (2000). *Modeling Survival Data: Extending the Cox Model.* New York, NY: Springer New York, 10.1007/978-1-4757-3294-8

[B49] Vandenheede M. Bouissou M. F. (1994). Fear reactions of ewes to photographic images. *Behav. Process.* 32 17–28. 10.1016/0376-6357(94)90024-8 24925110

[B50] Wemelsfelder F. Farish M. (2004). Qualitative categories for the interpretation of sheep welfare: a review. *Anim. Welf.* 13 261–268.

