# Supplementary figures and images for: Attention Bias Test Differentiates Anxiety and Depression in Sheep
Source: Front Behav Neurosci. 2018 Oct 23;12:246. doi: 10.3389/fnbeh.2018.00246 (PMC6205987; doi:10.3389/fnbeh.2018.00246)

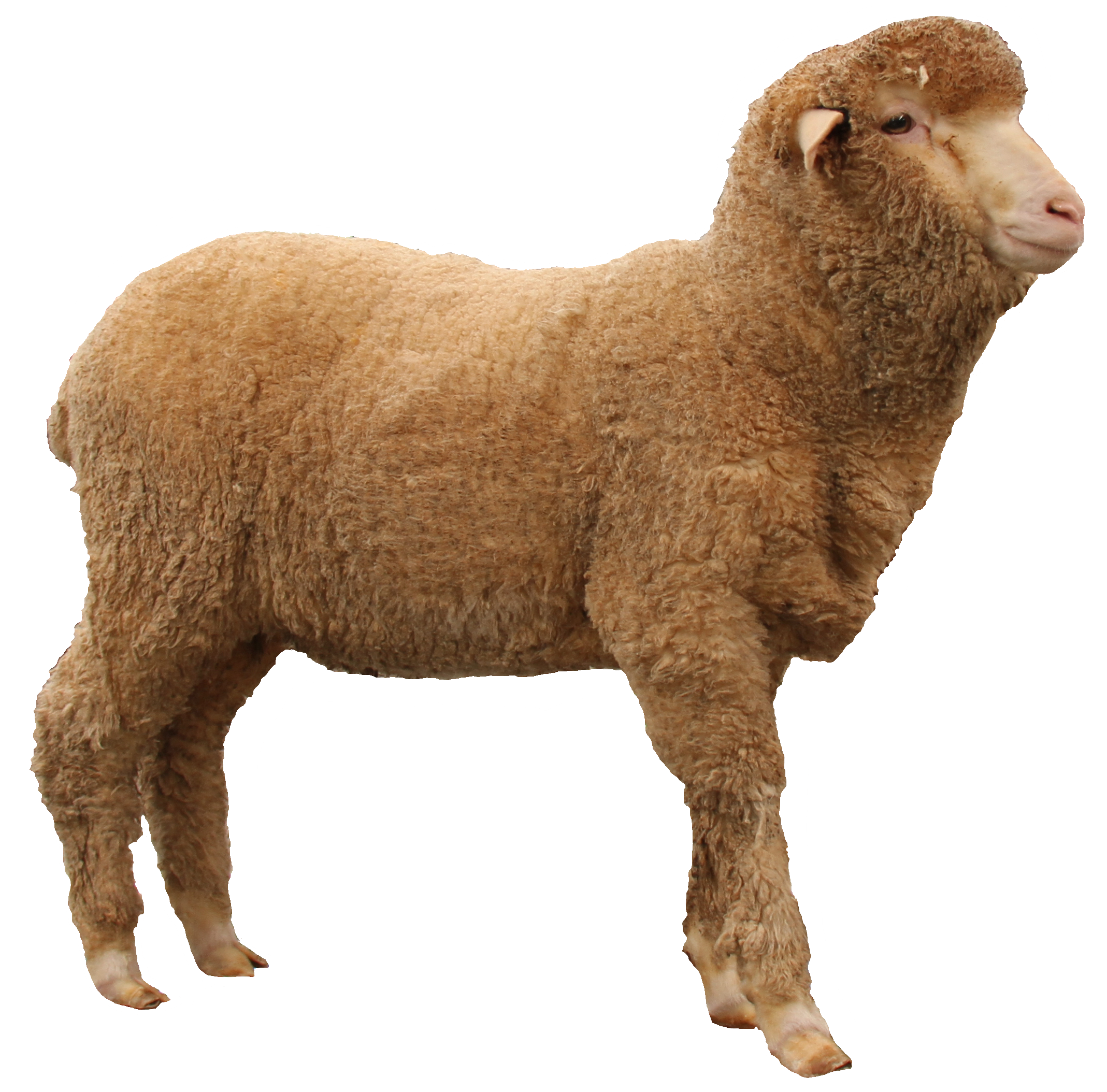

Supplement: FIGURE S1 — Photograph of a sheep in side-profile. The photograph was printed to be approximately life-size on A0, matte, 200 gsm cardstock then was cut out and mounted to 5 mm thick black corflute board using spray adhesive for use in the modified attention bias test. [file Image_1.PNG]
